# Dystrophin Deficiency Compromises Force Production of the Extensor Carpi Ulnaris Muscle in the Canine Model of Duchenne Muscular Dystrophy

**DOI:** 10.1371/journal.pone.0044438

**Published:** 2012-09-04

**Authors:** Hsiao T. Yang, Jin-Hong Shin, Chady H. Hakim, Xiufang Pan, Ronald L. Terjung, Dongsheng Duan

**Affiliations:** 1 Department of Biomedical Sciences, College of Veterinary Medicine, The University of Missouri, Columbia, Missouri, United States of America; 2 Department of Molecular Microbiology and Immunology, School of Medicine, The University of Missouri, Columbia, Missouri, United States of America; University of Minnesota, United States of America

## Abstract

Loss of muscle force is a salient feature of Duchenne muscular dystrophy (DMD), a fatal disease caused by dystrophin deficiency. Assessment of force production from a single intact muscle has been considered as the gold standard for studying physiological consequences in murine models of DMD. Unfortunately, equivalent assays have not been established in dystrophic dogs. To fill the gap, we developed a novel *in situ* protocol to measure force generated by the extensor carpi ulnaris (ECU) muscle of a dog. We also determined the muscle length to fiber length ratio and the pennation angle of the ECU muscle. Muscle pathology and contractility were compared between normal and affected dogs. Absence of dystrophin resulted in marked histological damage in the ECU muscle of affected dogs. Central nucleation was significantly increased and myofiber size distribution was altered in the dystrophic ECU muscle. Muscle weight and physiological cross sectional area (PCSA) showed a trend of reduction in affected dogs although the difference did not reach statistical significance. Force measurement revealed a significant decrease of absolute force, and the PCSA or muscle weight normalized specific forces. To further characterize the physiological defect in affected dog muscle, we conducted eccentric contraction. Dystrophin-null dogs showed a significantly greater force loss following eccentric contraction damage. To our knowledge, this is the first convincing demonstration of force deficit in a single intact muscle in the canine DMD model. The method described here will be of great value to study physiological outcomes following innovative gene and/or cell therapies.

## Introduction

The absence of dystrophin leads to Duchenne muscular dystrophy (DMD), an incurable lethal muscle disease that affects approximately 1 to 3 boys per every 10,000 male births [1], [2], [3]. The dystrophin gene was discovered in 1987 by Kunkel and colleagues [4], [5]. It was soon determined that dystrophin scaffolds a series of cytosolic and trans-membrane proteins into the dystrophin-associated glycoprotein complex (DGC). A major cellular function of the DGC is to crosslink the cytoskeleton with the extra cellular matrix and protects the sarcolemma from mechanical injuries generated during muscle contraction.

With the knowledge of the dystrophin gene, two naturally occurring dystrophin-null animals were soon identified. These are mdx mice and golden retriever muscular dystrophy (GRMD) dogs [6], [7], [8], [9]. Dystrophin expression is abolished in these animals due to point mutations in the dystrophin gene. Both mdx mice and GRMD dogs show characteristic muscle pathology including degeneration/regeneration, necrosis, fibrosis and inflammation. Interestingly, young adult mdx mice appear quite healthy and they manifest minimal dystrophic symptoms. In striking contrast, young adult dogs are clinically crippled and display apparent muscle wasting and loss of mobility.

The phenotypic resemblance between dystrophic dogs and human patients suggests that dystrophin-null dogs may serve as a more relevant preclinical model [10], [11], [12], [13], [14]. Further, the body size similarity between dogs and affected children may offer additional advantages in translation. Unfortunately, few studies have been performed in the canine model [14]. A factor that has limited the use of the dog model is the lack of robust outcome measurements. In particular, no assay has been established to allow reliable determination of force changes in a single intact muscle in dystrophic dogs. Studying effect in a single intact muscle represents a logical approach at the early stage of new therapy development. As a matter of fact, the proof-of-principle for essentially every ongoing genetic therapy was originally demonstrated in a single intact muscle in mdx mice. Some of these examples include adeno-associated virus (AAV) mediated micro/mini-dystrophin gene replacement therapy and anti-sense oligonucleotides mediated exon skipping. Corroboration of promising murine results in the dog model has now become a rate-limiting factor in the development of novel DMD therapies. So far, the contractile profile of dystrophic dogs has only been studied using whole hind limb preparation which measures force generated from a group of muscles rather than a single discrete muscle [15]. Attempts to delineate contraction deficiency in a single intact muscle in dystrophic dogs have not been very successful [16]. Kornegay and colleagues measured force in the peroneus longus muscle of GRMD dogs. Characteristic dystrophic muscle pathology was observed. However, the authors did not detect a significant difference in muscle weight normalized forces between normal and affected dogs, nor was a cardinal feature of muscle deficit, susceptibility to eccentric contraction examined [16].

To meet the need of preclinical translational studies, here we explored whether a robust and sensitive physiology assay can be developed to faithfully study contraction defects in a single intact muscle in dystrophic dogs. *In vitro* force measurement in the extensor digitorum longus (EDL) muscle and *in situ* force assay in the tibialis anterior (TA) muscle are two most frequently used methods in murine studies [17]. Considering the size of the muscle (a large muscle cannot be fully oxygenated *in vitro*) and the accessibility for therapeutic agent delivery (e.g. gene or cell transfer), we decided to focus on the extensor carpi ulnaris (ECU) muscle and an *in situ* force measurement approach. We examined dystrophin expression, histopathology, tetanic muscle force, fatigue and eccentric contraction profiles in seven normal and seven affected dogs. Characteristic pathological lesions were observed in dystrophin-deficient ECU muscles. Compared to that of wild type controls, dystrophic dogs showed a significant reduction in both absolute and specific tetanic forces. During isometric contractions there was no difference in the fatigue profile between two groups. Importantly, however, affected dogs showed a much larger force decline upon repeated cycles of eccentric contraction challenge.

## Materials and Methods

### Animals

**Figure 1:**
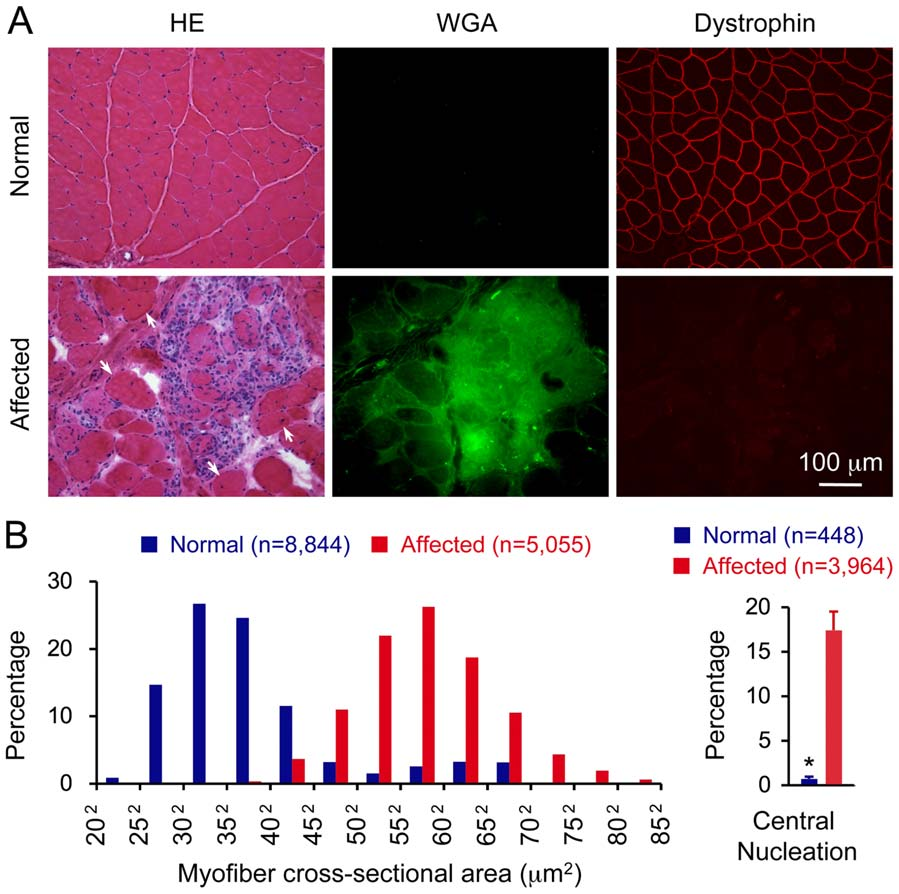
Dystrophin deficiency leads to severe muscle damage in the extensor carpi ulnaris muscle in affected dogs. A, Haematoxylin and eosin (HE) staining (left panel), wheat germ agglutinin (WGA) staining (middle panel) and dystrophin immunofluoresence staining (right panel) of the extensor carpi ulnaris muscle from a normal dog (top panel) and an affected dog (bottom panel). WGA staining reveals tissue fibrosis. Arrow, regenerative myofibers with centrally located nuclei. **B,** Morphometric quantification of the myofiber size (left panel) and central nucleation in normal and affected ECU muscles. The myofiber size is expressed according to the distribution of the cross-sectional area of individual myofibers. Results for the normal ECU muscle was from 8,844 myofibers of five normal dogs. Results for the dystrophic ECU muscle was from 5,055 myofibers of four affected dogs. The central nucleation is expressed as the percentage of myofiber with centrally localized nuclei among all myofibers counted. Results for the normal ECU muscle was from 9,467 myofibers of five normal dogs. Results for the dystrophic ECU muscle was from 3,964 myofibers of four affected dogs. Asterisk, significantly different (p  = 0.0001).

All animal experiments were approved by the Animal Care and Use Committee of the University of Missouri (# 6739) and were performed in accordance with NIH guidelines. Experimental dogs were produced at the University of Missouri by artificial insemination. These dogs were on a mixed genetic background of golden retriever, labrador retriever, welsh corgi and beagle. Affected dogs were initially diagnosed by elevated creatine kinase levels and PCR genotyping using our published protocols [18], [19]. The diagnosis was further confirmed by dystrophin immunofluorescence staining (see below) (Figure 1). Age and body weight of experimental dogs are shown in Table 1.

**Table 1:** Animal information and muscle data.

	Normal	n	Affected	n	P value
Age (year)	1.64±0.30	7	1.61±0.30	7	0.944
Body weight (Kg)	20.34±2.76	7	16.57±1.55	7	0.256
ECU muscle					
Muscle weight (g)	8.37±1.83	7	6.08±0.93	7	0.287
Weight ratio (g/kg body weight)	0.39±0.04	7	0.36±0.03	7	0.604
Muscle length (cm)	14.48±1.40	7	16.04±0.81	7	0.354
Fiber length (cm)	0.65±0.06	7	0.72±0.04	7	0.354
PCSA (cm^2^)	11.37±1.70	7	7.68±0.82	7	0.074
Tetanic force					
Absolute (N)	97.15±16.40	7	54.94±7.07	7	0.034
Specific, per muscle weight (N/g)	12.45±0.82	7	9.21±0.54	7	0.006
Specific, per PCSA (N/cm^2^)	8.38±0.41	7	7.04±0.36	7	0.031
Eccentric contraction parameters					
Maximal tension (N)	282.76±22.68	3	204.84±59.21	4	0.332
% Length stretched	5.00±0.00	3	4.97±0.02	7	0.360
Stretch rate (mm/s)	7.90±1.15	3	7.14±0.65	7	0.500

ECU, extensor carpi ulnaris muscle.

n, number of dogs studied.

N, Newton.

PCSA, physiological cross-sectional area.

P value obtained from unpaired Student T test.

### Morphology Studies

General muscle histopathology was revealed with haematoxylin and eosin (HE) staining. Muscle fibrosis was evaluated by immunofluorescence staining with the wheat germ agglutinin (WGA)-Oregon Green conjugate (Molecular Probes, W6748; 5 µg/mL PBS). Fibrotic tissues stain in green color. Dystrophin was examined by immunofluorescence staining with a monoclonal antibody against the dystrophin C-terminal domain (Dys-2, 1∶30 dilution, Novocastra, Newcastle, UK). Immunofluorescence staining was performed according to our previously published protocol [20], [21]. The percentage of centrally nucleated myofibers were quantified in HE stained muscle cross-sections as we described before [18]. The cross-sectional area of individual myofiber was determined from the digitized images using the quantitative image analysis module of the extended version of the Photoshop CS5.5 software (Photoshop version CS5.5 extended, Adobe Systems Incorporated, San Jose, CA). Photomicrographs were taken with a Qimage Retiga 1300 camera using a Nikon E800 fluorescence microscope.

### Muscle Function Evaluation

#### Surgical preparation

The experimental subject was sedated with ketamine (15 mg/kg body weight) and acepromazine (0.12 mg/kg body weight). Anesthesia was induced with 4% isoflurane. The subject was then intubated with an endotracheal tube connected to a mechanical ventilator (Ohmeda 7000, Ohmeda, Madison, WI). The breath rate was set at 15–18 per min and the tidal volume was set at 10 ml/min/kg body weight. During the experiment, anesthesia was maintained with 2% isoflurane and 98% oxygen.

Body hair in the surgical area was shaved. The right carotid artery and jugular vein were surgically exposed. A catheter was inserted into the carotid artery and advanced to the thoracic aorta for blood pressure monitoring. Another catheter was inserted into the jugular vein for intravenously lactated saline infusion. Core body temperature (rectal temperature), blood pressure and electrocardiograph (including the heart rate) were monitored throughout the experiment.

The dog was placed in a supine position on a force transducer plate that was specially designed for *in situ* muscle function assay. A 3 cm incision was made at the medial side of the upper forelimb. The brachial artery was exposed and a 3P transonic flow probe was put around the brachial artery for blood flow measurement (Module TS 420, Transonic Systems, Ithaca, NY). Another incision was made on the lateral side of the forearm to expose the entire ECU muscle. The length of the entire ECU preparation (muscle plus tendon) was measured as the distance from the proximal tendon insertion at the medial epicondyle of the humerus to the distal tendon insertion at the carpus. The length of the tendons (proximal and distal) accounts for 16% of the length of a complete ECU preparation (muscle plus tendon) (Yang, Shin, Terjung and Duan, unpublished observation). The length of the experimental ECU muscle was calculated by subtracting the tendon length (16% of the total length) from the total measured length (Table 1). The distal ECU tendon was cut at the insertion on the carpus. The free end of the distal tendon of ECU muscle was then sewn on a metal washer (5/16 size) with #2 surgical silk. The washer was tightly held by two metal blocks each with a U shaped groove in the middle to let the muscle tendon pass through. The metal block was fastened with two screws onto the force transducer (SM-250–38, Interface, Scottsdale, AZ). The forearm was subsequently fixed with two bone pins to allow the ECU muscle in line with the muscle force transducer. One stainless steel bone pin was placed on the olecranon and the other was placed on the radius about 3 cm away from its distal end. The radial nerve was located at the lateral side of the distal humerus bone. To expose the radial nerve for electric stimulation, the forearm incision was slightly extended proximately until the nerve was clearly visible. The radial nerve was carefully dissected and tied. The nerve was then cut and its distal end was mounted on a bipolar electrode for triggering muscle contraction. Radial nerve stimulation resulted in contraction of the whole extensor muscle groups. However, since the force transducer was only connected to the ECU muscle tendon, only the force produced by the ECU muscle was recorded. It should be pointed out that since the forelimb was tightly held by two strong bone pins (one on the olecranon and the other on the radius), radial nerve stimulation did not cause any movement of the forelimb except for foot kicking due to extensor muscle contraction. To determine whether foot kicking affected tension measurement, we compared the results with or without foot fixation. We did not see any difference in the recorded tension. The exposed ECU muscle and tendon were moistened with warm (37°C) saline gauze. The temperature of the ECU muscle surface was maintained at 37°C with a surgical lamp. After the preparation was done, the subject was allowed to stabilize for 10 minutes before force measurement. In some subjects, the same experiment was conducted on both sides of the forelimb. At the end of study, the subject was euthanized and necropsied.

#### Force measurements

**Figure 2:**
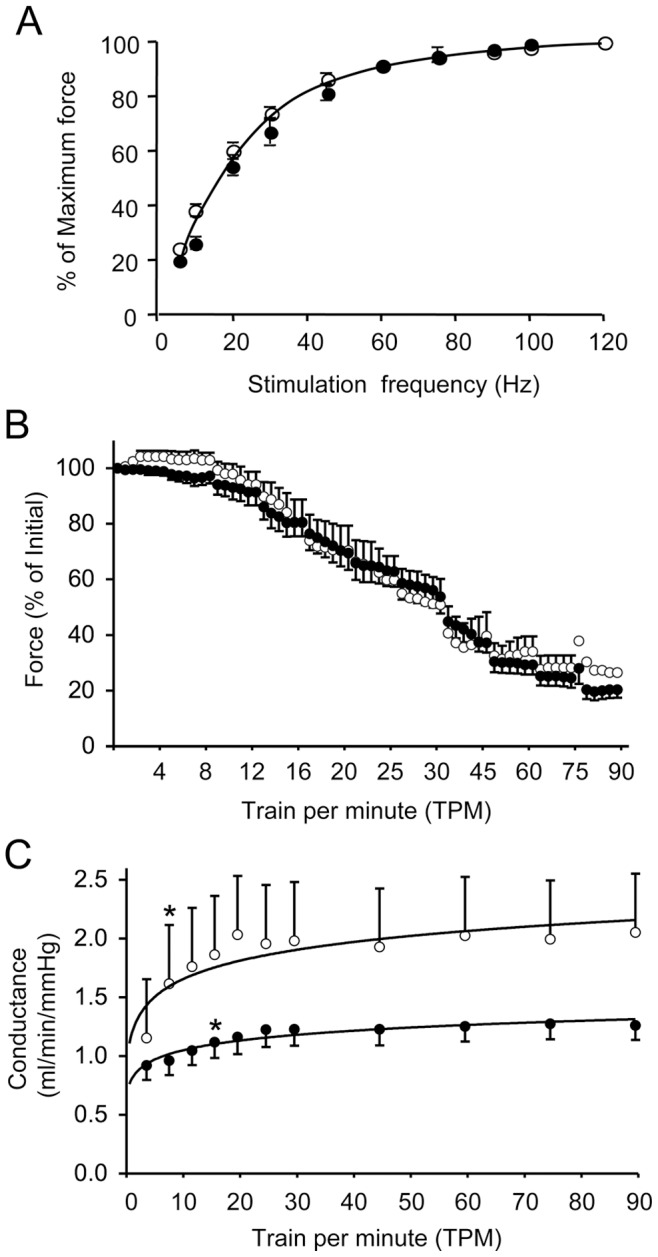
Force-frequency relationship in the ECU muscle and impact of repetitive tetanic contraction on muscle force and blood flow. A, Force-frequency relationship in the ECU muscle of normal and dystrophin-null dogs. Single tetanic force at different stimulation frequency was recorded under optimal muscle length (L_0_). After normalizing to muscle weight, relative tetanic force at each stimulation frequency was calculated as the percentage of the maximal single tetanic force (Table 1). Relative tetanic force was then graphed against stimulation frequency. Data are presented as mean ± standard error of mean. Open circle, normal dogs (n = 4). Closed circle, dystrophic dogs (n = 7). B, Force profile during continuous tetanic stimulation. Uninterrupted stimulation was started at a train frequency of 4 TPM. The train frequency increased every 5 min to 8, 12, 16, 20, 25, 30, 45, 60, 75 and finally 90 TPM. Muscle force was recorded every minute. The tetanic force prior to the start of train stimulation was defined as 100%. Subsequent force change was compared accordingly and expressed as the percent of the starting tetanic force. Open circle, normal dogs (n = 4). Closed circle, dystrophic dogs (n = 7). C, Forearm blood flow conductance (ml/min per mmHg) during continuous tetanic stimulation. Logarithm trend lines are depicted for both normal and affected dogs. Open circle, normal dogs (n = 4). Closed circle, dystrophic dogs (n = 7). Asterisk, significantly different from that of pre-contraction (0 TPM) in the same group. No statistically significant difference was noted between normal and affected dogs by repeated measures analysis of variance (0.1>p>0.05).

For all experiments, electric stimulation was set at 8 volts and 0.2 ms pulse duration (Grass S48 Stimulator, Grass Instruments, Quincy, MA). Muscle force, brachial arterial blood flow and aorta blood pressure were recorded with Powerlab (AD Instruments, Castle Hill, Australia) interfaced with a Mac power PC computer. The optimal muscle length was determined using single twitch stimulation. Briefly, twitch stimulation was applied while the muscle was hold at different lengths. The muscle length which yielded the highest twitch force was defined as the optimal muscle length (Lo). At the optimal muscle length, the force-frequency relationship was determined by applying 200 ms tetanic stimulation at various stimulation frequencies (Figure 2A). The frequency that yielded the highest force (usually 90 to 100 Hz) was defined as the optimal stimulation frequency. The optimal muscle length (Lo) and optimal stimulation frequency were used in all subsequent force measurements. The peak tetanic force was determined as the highest force produced during 100 to 200 ms tetanic stimulation (Table 1).

#### Determination of the fiber length (Lf) to optimal muscle length ratio (Lf/Lo) in the ECU muscle

The freshly isolated ECU muscle was pinned at Lo with two 22G 1.5-inch needles on a piece of 4-inch thick Styrofoam board. The blood was rinsed off the muscle with the Ringer’s buffer. The muscle was then sutured to a piece of wood stick using 1–0 suture to fix it at the Lo. After 3 to 4 days’ fixation in 10% formaldehyde, the muscle was macerated in 15% nitric acid (HNO_3_) for five days. After 5 days of maceration in 15% nitric acid, the muscle was then transferred to 30% nitric acid for ∼30 min until the myofiber bundles became loosely attached to the muscle. The muscle was carefully rinsed with 50% glycerol several times and then stored in 50% glycerol. Approximately 10 to 20 myofiber bundles (∼5 to 10 intact myofibers per bundle) were gently teased from the proximal, middle and distal portions of the muscle under a dissection microscope. Individual intact myofiber was carefully separated from the bundle and digital images were taken under 2x magnification. The myofiber length was then measured with the Photoshop CS5.5 software. The fiber length of approximately 100 myofibers was measured for each ECU muscle from three normal dogs (16.22±4.32 month-old, mean ± sem) using the length measurement function of the NIH image J software (version 1.45h). The Lf/Lo ratio was 0.0448±0.0025 (mean ± sem). The value 0.0448 was used as the average Lf/Lo ratio for physiological cross-sectional area (PCSA) calculation.

#### Determination of the PCSA of the ECU muscle

The ECU muscle is a bi-pennate muscle. Its PCSA is proportional to the cosine of the pennation angle [22], [23], [24]. We first measured the pennation angle from the ECU muscles of three normal dogs (the same dogs used for Lf/Lo ratio determination) [25]. The pennation angle was 10.03±0.80 (mean ± sem). The value 10.03 was used as the average pennation angle for PCSA calculation. The PCSA (cm^2^) of each muscle was calculated according to the equation PCSA  =  (muscle weight in gram x cos10.03)/(1.056 g/cm^3^ x optimal fiber length in cm). 1.0597 g/cm^3^ is the muscle density [26]. The optimal fiber length was determined by multiplying the measured Lo of the muscle by the Lf/Lo ratio of 0.0448.

#### Determination of the specific muscle force

The specific muscle force was calculated by normalizing the tetanic muscle force by the PCSA. For comparison, we also determined the muscle weight normalized muscle force (Table 1).

#### Fatigue protocol

Muscle was rested for 1 min. Resistance to fatigue was then tested by applying trains of repeated 200 ms tetanic stimulation at gradually increasing train frequencies of 4, 8, 12, 16, 20, 25, 30, 45, 60, 75, and 90 tetani per min (TPM). Muscle was stimulated for 5 minutes at each train frequency and immediately shifted to a higher train frequency without interruption.

#### Eccentric contraction protocol

After the fatigue protocol, the ECU muscle was rested for 20 min. The peak force was recovered by 95±10.6% in normal dogs, and 90±4.6% in affected dogs. The ECU muscle was then subjected to eccentric contraction. The stimulation parameters were the same as described above (8 volts, 0.2 ms pulse duration, optimal muscle length and optimal stimulation frequency). In each cycle of eccentric contraction, the ECU muscle was stimulated for a total of 1200 msec. No stretch was performed during the first 100 msec to allow collection of the tetanic force data. In subsequent 1100 msec, the ECU muscle was stretched manually by ∼ 5% of its original length. Briefly, muscle stretch was established manually by uniform rotation of a large screw-thread-knob to achieve a linear withdrawal of the force transducer, with the force and length recorded over time (Powerlab). This record was used to calculate the actual stretch length (mm/ECU muscle length) and uniform stretch rate (mm/sec). The details of the stretching parameters are listed in Table 1. After a 2 min rest, a second cycle of eccentric contraction was applied. A total of ten cycles of eccentric contraction were conducted in each ECU muscle. The tetanic force generated at the beginning of the first cycle eccentric contraction was defined as 100%. The tetanic forces obtained in each subsequent cycle were used to calculate force drop induced by eccentric contraction. The percentage of force drop was determined according to the formula, force drop %  = 100×(T1–Tn)/T1, where T1 stood for the tetanic force obtained during the first cycle and Tn represented the tetanic force obtained during the nth cycle.

### Blood Flow

Blood flow rate (ml/min) in the brachial artery and aortic blood pressure were recorded throughout the entire experiment. To more accurately reflect blood flow change during repeated tetanic contraction, we calculated blood flow conductance using the formula, conductance  =  blood flow rate (ml/min)/blood pressure (mmHg).

### Statistical Analysis

Data are presented as mean ± standard error of mean. For comparison of two groups, unpaired Student t test was applied. For muscle performance, blood pressure and blood flow data, repeated measures one way analysis of variance was applied. This analysis allowed determination of the effect of muscle disease (muscular dystrophy) and muscle contraction as well as the interaction between muscle disease and muscle contraction. Tukey’s test was used for *post hoc* analysis. P<0.05 was considered statistically significant.

## Results

A total of seven normal and seven affected dogs were studied. There was no statistically significant difference in body weight and age between normal and affected dogs (Table 1). Normal dog ECU muscle showed characteristic sarcolemmal dystrophin expression (Figure 1). As expected, dystrophin was not detected in the ECU muscles obtained from affected dogs (Figure 1). ECU muscle pathology was examined by HE staining. Normal dog muscle had uniform fiber size, peripherally located myonuclei and there was minimal inflammatory cell infiltration. Affected dogs showed prominent muscle degeneration, necrosis, myofiber size variation and inflammation (Figure 1). More then 15% of myofibers contained centrally localized nuclei in affected dogs while it was only less than 1% in normal dogs (Figure 1). The distribution of the cross-sectional area of individual myofibers was also altered in dystrophic dogs (Figure 1). To evaluate muscle fibrosis, we performed WGA immunostaining. Only affected dog ECU muscle showed pronounced fibrosis (Figure 1).

Force from ten normal and eight affected ECU muscles was examined. These include ten ECU muscles from seven normal dogs and eight ECU muscles from seven affected dogs. In four normal dogs, force was measured from only one ECU muscle. In the other three normal dogs, force was measured from both sides. In six affected dogs, force was measured from only one ECU muscle. In one affected dog, force was measured from both sides) (Table 1). In cases where both side ECU muscles were studied, force from the left and right ECU muscles were combined as the data from one dog for statistical comparisons. There was no significant difference in body weight, muscle weight, muscle length, fiber length and PCSA between normal and affected dogs (Table 1). Force-frequency relationship was not altered by dystrophin deficiency (Figure 2A). The half relaxation time was 29.7 ± 1.68 ms in normal group and 29.2 ± 0.92 ms in affected group. There was no significant difference between normal and affected dogs (p>0.05). Absolute muscle force and specific tetanic force (normalized either by muscle weight or PCSA) were significantly reduced in affected dogs (Table 1).

To evaluate muscle fatigue, we applied continuous tetanic contractions at the rate of 4 to 90 TPM (Figure 2B). A similar pattern was observed in both normal and affected dogs. No loss of initial tension was observed up to 16 TPM. At the end of 90 TPM contractions, ∼25% of initial force was still retained in both groups (Figure 2B).

We also measured the change of brachial blood flow during repeated tetanic contraction (Figure 2C). Blood flow increased as the frequency of the train stimulation increased from 4 to 30 TPM. Statistic analysis by ANOVA suggests that there is a strong correlation between contraction and blood flow increase (p<0.001). Interestingly, the rate of conductance increase in normal dogs was 67% higher than that of affected dogs. For normal dogs, the slope before reaching plateau was 0.10 ± 0.04. For affected dogs, the slop was 0.06 ± 0.02 (p<0.1) (Figure 2C). After 30 TPM, blood flow reached plateau and no further increase was noted.

**Figure 3:**
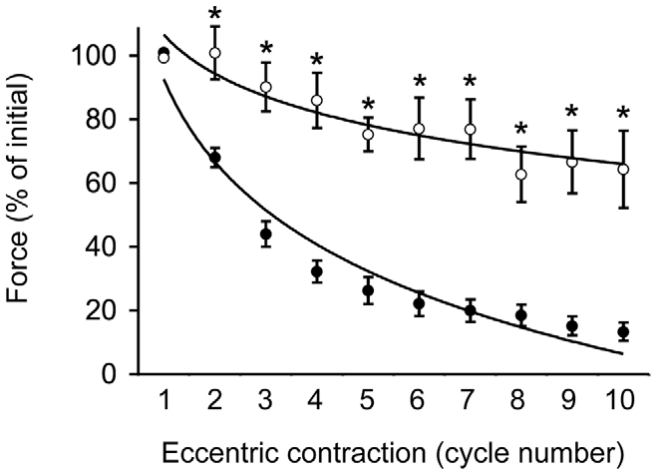
The ECU muscle of affected dogs was more vulnerable to eccentric contraction induced damage. Relative changes of the tetanic force before the start of eccentric contraction and after each cycle during 10 cycles of eccentric contraction. The force at the beginning of the first cycle of eccentric contraction was designated as 100%. Logarithm trend lines are depicted for both normal and affected dogs. Open circle, normal dogs (n = 3). Closed circle, dystrophic dogs (n = 7). Asterisk, significantly different from that of affected dogs.

To characterize contraction-induced muscle damage, we challenged the ECU muscle with 10 cycles of eccentric contraction (Figure 3, Table 1). The ECU muscle was stretched while it underwent tetanic contraction. Similar stretch parameters (maximal tension, percentage of length stretched and the stretch rate) were applied to normal and affected dogs (Table 1). In normal dogs, no significant force reduction was observed until the sixth cycle of eccentric contraction. Even after all 10 cycles of eccentric contraction, the normal ECU muscle still maintained ∼64% of the initial force (Figure 3). In sharp contrast, a dramatic force drop was observed even after the first cycle of eccentric contraction in affected dogs (p<0.01). By the end of the 10^th^ cycle of eccentric contraction, tetanic force reduced to only ∼12% of the initial force in the dystrophic ECU muscle (Figure 3). Statistic analysis by ANOVA showed a strong interaction (p<0.01) of dystrophin deficiency and eccentric contraction in force decline. Blood pressure and brachial blood flow showed no statistical difference (either within the same group or between groups) during eccentric contraction.

## Discussion

A bottleneck in the development of DMD gene and/or cell therapy is the dog study. Despite consensus agreement on the importance and relevance of the canine model, it remains a great challenge to convincingly demonstrate physiological benefits of treatment in dystrophic dogs. Studies conducted so far have been mainly focused on dystrophin expression and morphological amelioration [27], [28], [29]. Definitive force improvement data that are essential to buttress the microscopic findings are missing [14]. Even worse, a solid protocol has not been developed to demonstrate force deficiency in a single intact muscle in dystrophin-deficient dogs, not to mention quantifying variable levels of (potentially subtle) improvements incurred by experimental therapy. A major goal of the current study is to fill this knowledge gap and establish a rigorous and sensitive assay to evaluate contractility of a single intact dog muscle. This platform can then be used to test functional outcome of any gene/cell therapy strategies in the canine model.

We first determined which muscle to use. We considered the following factors in decision making. These are (1) whether the muscle can be readily approached and fully saturated by local injection, (2) whether the muscle have defined anatomic structure (such as a long slender tendon) for *in situ* mounting on the force transducer plate and (3) muscle pathology. A preliminary study was performed on the gastrocnemius, extensor carpi radialis and ECU muscles (data not shown). The results suggest that the ECU muscle is an ideal target muscle for our purpose. The ECU muscle can be easily identified based on the surface marks. Saturated delivery can be achieved in the ECU muscle via surgical or percutaneous injection (data not shown) [28]. This muscle also showed characteristic muscle pathology and yielded consistent force data in dogs of different size and breeds (Figure 1).

The following major findings were made. First, we observed a significant reduction of tetanic force in affected dogs (Table 1). In contrast to what have been reported for the peroneus longus muscle [16], the ECU muscle of dystrophin-deficient dogs uniformly showed a significantly reduced specific isometric tetanic muscle force (either normalized by muscle weight or PCSA) (Table 1). As a consequence of muscle degeneration and necrosis, a loss of specific isometric force is expected in dystrophin-deficient muscle. However, this has only been shown in mdx mice. Our results here provide direct evidence that specific force reduction also presents in large mammals that carry a null mutation in the dystrophin gene.

The second major finding is on eccentric contraction. We observed a much greater force decline in affected ECU muscles. A statistically significant difference was obtained even after one cycle of eccentric contraction (Figure 3). A major function of dystrophin is to protect muscle cells from contraction-induced injury. Forcibly lengthening of an actively contracted muscle is one of the most sensitive and stringent physiological tests of muscle integrity [30]. The degree of immediate force drop after eccentric contraction is now a widely accepted index for evaluating the therapeutic efficacy of an experimental DMD therapy in the murine model. Immediate force changes after eccentric contraction have not been evaluated in the DMD dog model until recently [31]. Tegeler et al tested the effect of eccentric contraction using a tibiotarsal joint preparation. This preparation measures the collective effect from all muscles in the cranial tibial compartment. After 30 cycles of eccentric contraction, normal and affected dogs lost 8% and 63% of their initial torque, respectively [31]. The authors concluded that the flexor muscles of dystrophic dogs induced a greater force drop than that of normal dogs [31]. Our results here have confirmed and extended the study by Tegeler et al and suggest that the extensor muscle is also quite sensitive to lengthening contraction injury in dystrophic dogs. It is worth to point out that the method reported by Tegeler et al measures cumulative responses from many muscles and it cannot distinct the contribution of individual muscle. The ability to measure force alteration in a single dog muscle has significant advantages in translational studies. It provides a convenient avenue to compare different therapeutic strategies before launching much more expensive whole limb or whole body therapy. This is especially relevant in the case of AAV micro-dystrophin vectors. More than a dozen different constructs have been described (reviewed in [14], [32], [33]). A side-by-side comparison in a single dog muscle will be cost-effective to prioritize these microgene vectors. Another advantage of the single muscle approach is the possibility of correlating transduction efficiency and force improvement in the same muscle. It is currently not clear what percentage of myofibers should be transduced in order to see a physiological improvement in dystrophin-null large mammals. The single muscle assay platform described here may allow investigators to achieve this goal by titrating vector dose, viral serotype and injection protocol (such as the volume and speed etc).

Besides specific tetanic force and eccentric contraction, we also examined the fatigue profile and blood perfusion during tetanic muscle contraction. Similar to what have been reported in the limb muscle of mdx mice [34], [35], the ECU muscle from normal and affected dogs showed the same fatigue profile (Figure 2B). An important role of dystrophin is to anchor neuronal nitric oxide synthase (nNOS) to the sarcolemma [36]. Nitric oxide produced by membrane-associated nNOS counteracts sympathetic vasoconstriction in contracting muscle. In the absence of dystrophin, this protective mechanism is compromised [37], [38]. The resulting functional ischemia is now considered as a crucial pathogenic mechanism for DMD. In agreement with this theory, we indeed found a blunted (though not statistically significant) blood flow in affected dogs when the ECU muscle was subject to continuous tetanic stimulation (Figure 2C). Future studies are needed to further corroborate this exciting preliminary observation.

The reduction of specific muscle force and the increase of susceptibility to eccentric contraction injury are the most consistent findings in the murine DMD model (reviewed in [39]). Our results demonstrated for the first time that a single intact muscle from dystrophin-null dogs share the same features. A straining issue in the use of the dog model is the lack of rigorous physiological methods to objectively evaluate the outcome. The *in situ* protocol described here, though a terminal assay, may therefore serve as a highly informative endpoint to accurately measure muscle force improvement in dog studies in the future.
